# A Computational Model to Predict Rat Ovarian Steroid Secretion from *In Vitro* Experiments with Endocrine Disruptors

**DOI:** 10.1371/journal.pone.0053891

**Published:** 2013-01-11

**Authors:** Nadia Quignot, Frédéric Y. Bois

**Affiliations:** 1 Chair of Mathematical Modeling for Systems Toxicology, Bioengineering Department, Université de Technologie de Compiègne, Compiègne, France; 2 Models for Ecotoxicology and Toxicology, Institut National de l’Environnement industriel et des Risques, Verneuil-en-Halatte, France; University of Cincinnati College of Medicine, United States of America

## Abstract

A finely tuned balance between estrogens and androgens controls reproductive functions, and the last step of steroidogenesis plays a key role in maintaining that balance. Environmental toxicants are a serious health concern, and numerous studies have been devoted to studying the effects of endocrine disrupting chemicals (EDCs). The effects of EDCs on steroidogenic enzymes may influence steroid secretion and thus lead to reproductive toxicity. To predict hormonal balance disruption on the basis of data on aromatase activity and mRNA level modulation obtained *in vitro* on granulosa cells, we developed a mathematical model for the last gonadal steps of the sex steroid synthesis pathway. The model can simulate the ovarian synthesis and secretion of estrone, estradiol, androstenedione, and testosterone, and their response to endocrine disruption. The model is able to predict ovarian sex steroid concentrations under normal estrous cycle in female rat, and ovarian estradiol concentrations in adult female rats exposed to atrazine, bisphenol A, metabolites of methoxychlor or vinclozolin, and letrozole.

## Introduction

Humans may be exposed to numerous chemicals that impact endocrine activity, and notably alter androgen/estrogen balance [1]. Among environmental chemicals, atrazine, vinclozolin, methoxychlor, and bisphenol A were found to be of particular concern. Atrazine, a triazine herbicide which has been widely used in agriculture and is persistent in surface water, has been described in several *in vitro* studies to increase estrogen through elevation of aromatase levels and activity [2], [3]. The fungicide vinclozolin has been documented for the anti-androgenic activity of its metabolite M2 *in vitro* [4] and *in vivo* [5]. Methoxychlor is an organochlorine pesticide of known estrogenic activities *in vitro* and *in vivo* [6]; its metabolite 2, 2-bis-(p-hydroxyphenyl)-1, 1, 1-trichloroethane (HPTE) displays estrogenic, anti-estrogenic, and anti-androgenic capacities *in vitro* [7]. Bisphenol A, a plasticizer, was clearly defined as an estrogenic agent due to its capacity to bind estrogen receptor with an EC50 in the sub-micromolar range [8]. As far as drugs are concerned, a good example of pharmacologically-designed endocrine modifier may be letrozole [9]. This potent and highly specific nonsteroidal competitive aromatase inhibitor, used for estrogen-dependent breast cancer, has been characterized by a half maximal inhibitory concentration (IC50) of 7 nM [10].

A potential target for endocrine disrupting chemicals (EDCs) is steroidogenesis. In females, sex steroids are synthesized primarily in the ovaries and derived from cholesterol through a series of biochemical reactions [11]. Among steroidogenic enzymes, cytochrome P450 aromatase (Cyp19), which catalyses the final irreversible conversion of androgens to estrogens in granulosa cells (GCs), appears to be a key target. Aromatase disruption is often associated with EDC toxicity [12], and several assay guidelines recommend testing chemicals for that endpoint [13]. Aromatase expression is regulated by follicle-stimulating hormone (FSH), through multiple signaling pathways including cyclic adenosine monophosphate (cAMP)-dependent regulatory events [14]. In GCs, the final steps of steroidogenesis are also mediated by 17β-hydroxysteroid-dehydrogenases (Hsd17b1 and Hsd17b2), which catalyze the conversion of inactive sex steroids to active ones *via* Hsd17b1 or *vice-versa* by Hsd17b2 [15].

Assessing EDC toxicity is a challenge, given the complexity of the endocrine system and despite the increasing development of data on its workings. Most standardized “regulatory” tests developed to study EDC toxicity involve rats. Those *in vivo* tests naturally integrate hormone metabolism and feedback loops. They typically look at relevant integrated toxicity endpoints, such as impact on fertility [16]. *In vitro* models have also been extensively developed: they are faster, cheaper, and they spare animal lives [17]. They help the researcher to elucidate toxic mechanisms in a simple isolated system and, when performed on human cells, they avoid difficult interspecies transpositions.

Both characterization and quantification of toxicity mechanisms are necessary for a reliable quantitative *in vitro* to *in vivo* extrapolation (QIVIVE) [18]. In order to improve QIVIVE for endocrine toxicity, we developed and parameterized a dynamic systems biology model of the final steps of steroidogenesis in rat ovaries. We calibrated our mathematical model in a Bayesian framework on the basis of *in vitro* experimental data obtained from rat granulosa primary cell cultures. For cross-validation, the *in vitro* model was transposed to an *in vivo* context and predictions were compared with *in vivo* hormone dosage data obtained in control animals. We finally used our model to predict the effects of five selected EDCs on gonad estradiol (E_2_) secretion, based on *in vitro* data following exposure to atrazine, bisphenol A, methoxychlor metabolite HPTE, vinclozolin metabolite M2, and letrozole. These chemicals were chosen based on their known endocrine activity *in vitro* and *in vivo*.

## Materials and Methods

### Test Chemicals

Atrazine (CAS number 1912-24-9, purity 97.1%) was provided by TCI Europe (Zwijndrecht, Belgium); methoxychlor (CAS number 72-43-5, purity >95%), HPTE (CAS number 2971-36-0, purity 97%), and bisphenol A (CAS number 80-05-7, purity 99%) were purchased from Sigma Aldrich Chemical Co. (Saint-Quentin-Fallavier, France); vinclozolin (CAS number 50471-44-8, purity 99.5%) was from Greyhound Chromatography (Birkenhead, UK); vinclozolin M2 (CAS 83792-61-4, purity >98%) was from Interchim (Montluçon, France).

### *In Vitro* Experiments

#### Rat GC isolation and *in vitro* culture

Immature (21 days old) Sprague-Dawley female rats (certified virus-free) were purchased from Janvier (Le Genest-Saint-Isle, France). They were housed with a 12 h light and 12 h dark cycle and received food and water *ad libitum*. All procedures were reviewed and approved by the Institutional Animal Care and Use Committee of INERIS. All animals were 26 days old at the start of treatment. Each animal was injected subcutaneously with diethylstilbestrol (DES; Sigma Aldrich Chemical Co., Saint-Quentin-Fallavier, France) dissolved in corn oil (100 mg/0.1 ml) every day for 3 days to increase the number of GCs. On the third day, the animals were sacrificed by a lethal intraperitoneal pentobarbital injection. Five animals were sacrificed for each experiment. The ovaries were harvested, and the associated fat, oviduct, and bursa ovary removed; the samples were placed in ice-cold medium 199 (M199; Sigma Aldrich), and punctured several times with a 26-gauge needle until the antral follicles ruptured and released the GCs. The GC-rich medium was centrifuged (200 *g*) for 5 min to obtain a GC pellet, which was resuspended in Dulbecco’s modified Eagle medium/Ham’s F-12 nutrient mix (DMEM/F-12; Sigma Aldrich) containing 5% fetal bovine serum, 100 µg streptomycin per ml, and 100 IU penicillin per ml. The cells (300,000/ml) were plated into 12-well culture plates (2 ml/well)), and grown at 37°C in a humidified atmosphere with 5% CO_2_. The cells were allowed to attach for 72 h prior to treatment to minimize any effects due to *in vivo* DES priming [19].

#### GC treatment

We performed two experimental studies: a baseline (control) study with measurements at 4 h, and an “EDC study” with control (0.1% dimethyl sulfoxide, DMSO, in serum-free and phenol red-free culture medium) and four chemicals (atrazine, bisphenol A, HPTE, and vinclozolin M2) at 10 µM in a final concentration of 0.1% DMSO culture medium, with measurements at 4 h. The chemical concentration was chosen on the basis of relevant literature [20]. Cellular viability was determined by trypan blue exclusion staining, visual inspection for morphology, and cellular attachment.

#### mRNA level and direct aromatase activity measurements

mRNA levels and direct aromatase activity were quantified according to previously described methods [20]. Briefly, mRNA was extracted from the cells then reverse transcribed. Target fragments were amplified by real-time polymerase chain reaction. Aromatase enzymatic activity was measured on microsomal fractions of GCs with the tritiated water release assay [21]. These experimental data were expressed as “fold difference” between treated and control conditions. Differences of single doses from controls were statistically analyzed with a Mann-Whitney non-parametric test. Differences with a *P* value of less than 0.05 were considered to be statistically significant.

### *In Vivo* Experiments

The female Sprague-Dawley rats used were approximately 8 weeks old at the start of chemical exposure. Estrous cycle staging was done with vaginal smears collected twice a day and classified microscopically as diestrus, proestrus, estrus, or metestrus [22]. We performed two experimental studies: a baseline (control) study, measuring ovarian steroid concentrations across the estrous cycle, and an “EDC study” where each animal in diestrus stage was administered a test chemical or vehicle by gavage (atrazine 200 mg/kg, dissolved in 0.5% methylcellulose; bisphenol A or methoxychlor at 200 mg/kg, dissolved in corn oil; vinclozolin 100 mg/kg, dissolved in corn oil). The animals were sacrificed six hours after treatment; ovaries were harvested, weighed, and homogenized in PBS-buffered water for tissue dosages. Atrazine, bisphenol A, methoxychlor metabolite HPTE, vinclozolin metabolite M2, testosterone (T), androstenedione (A), estrone (E_1_), and E_2_ were detected and quantified in whole ovaries by liquid chromatography with tandem mass spectrometry detection (LC–MS/MS) [23]. Differences between treated and control animals were statistically analyzed with a Mann-Whitney non-parametric test. Differences with a *P* value of less than 0.05 were considered to be statistically significant.

### Model Chemical

We choose to include an additional compound to further test and cross-validate our mathematical model. Letrozole appeared to be a very good choice, in the sense that it is pharmacologically designed to specifically inhibit aromatase, which is one of the main target described in our computational model. This compound was not tested on our *in vitro* and *in vivo* systems, but experimental data were gathered from the literature [10], [24].

### Mathematical Model Development

#### Model overview

**Figure 1:**
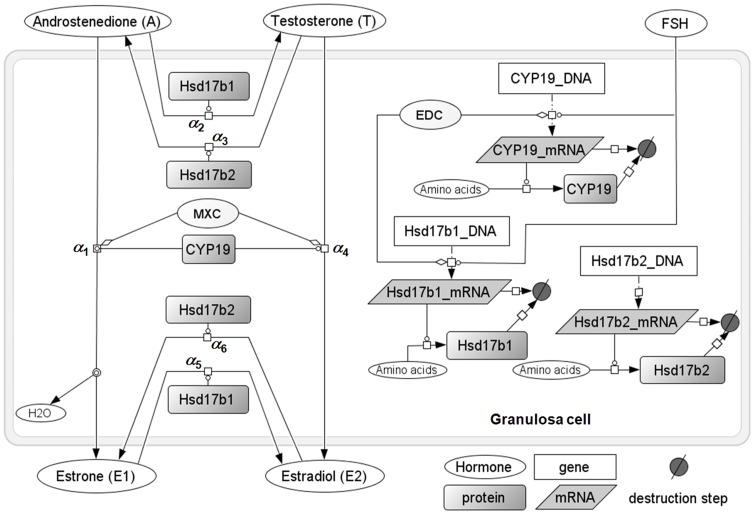
Overview of the computational model for steroidogenesis last metabolic steps in a rat granulosa cell. The transcription and translation events for the three last major enzymes involved in estradiol synthesis, and sex steroid synthesis itself, are modeled, with relevant FSH control, endocrine disrupting chemical (EDC) modulation, or methoxychlor (MXC) aromatase competitive inhibition. Steroids can be transported in and out of cell. *In vitro*, the exterior compartment corresponds to the culture medium; *in vivo* it corresponds to the ovary tissue (see Figure 2). Aliases (repeated species labels) are used for clarity but correspond in fact to a unique species.

**Figure 2:**
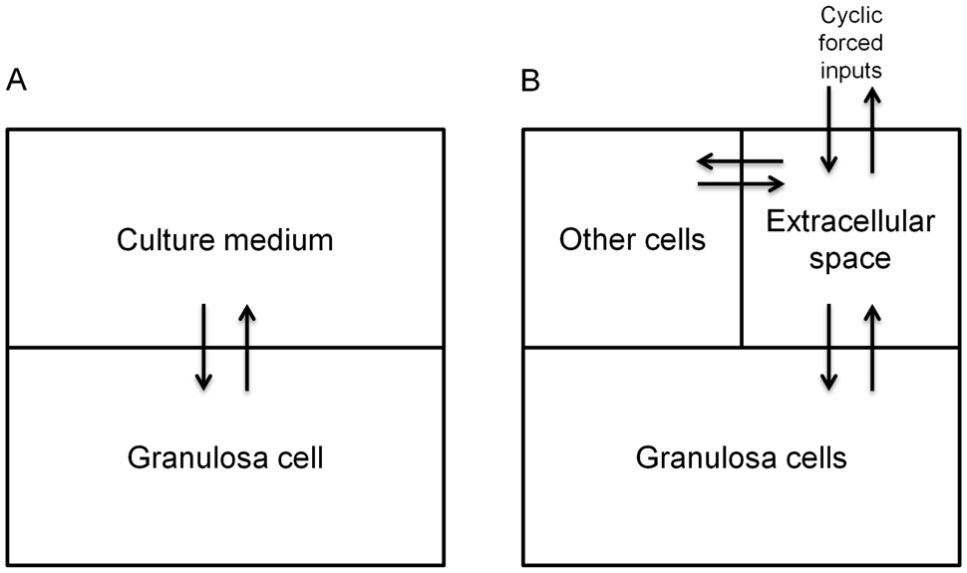
Overview of the compartments used to model *in vitro* (A) or *in vivo* (B) hormone transports. *In vitro* (A), the exterior compartment corresponds to the culture medium. *In vivo* (B), the ovary tissue is subdivided into three compartments: granulosa cells, “other cells” for thecal and interstitial cells, and extracellular space.

The model describes the final metabolic and transport steps of the steroidogenesis pathways in rat GCs (Figures 1 and 2). Metabolic steps include synthesis and degradation of Cyp19, Hsd17b1, and Hsd17b2 mRNAs and proteins, conversion of A into T, E_1_, and E_2_, and modulation of steroidogenic enzyme expression by FSH or an EDC. In vitro, transport includes GC uptake and secretion of A, T, E_1_, and E_2_. In vivo, transport also includes entry of A and T in ovaries, and exchange of hormones between extracellular space, GCs, and other kinds of cells (Figure 2).

#### Metabolic reactions

*mRNA and protein synthesis.* Cyp19 and Hsd17b1 mRNA quantities in GCs (*ε_mRNA_* in pg/cell, with *ε* = *Cyp19*, *Hsd17b1*, or *Hsd17b2*) depend on their synthesis with baseline rate *ν_mRNA,_ε* (pg/min). This rate is eventually altered by an EDC *X* (inducing fold-change *f_X_* (unitless)), upregulated by FSH (pg/cell) (with slope factors *κ* (/pg FSH), and affected by experimental variability (due to differences in cell pre-treatment, modeled by a variability factor *σ_L_* (arbitrary unit)); mRNA levels depend also on their degradation, with rate constant *δ_mRNA_* (/min):
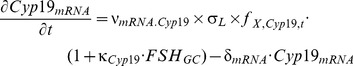
(1)

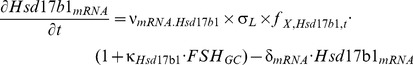
(2)


Fold-change for species *ε_mRNA_* was obtained from experimentally measured mRNA levels and computed as:
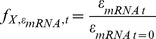
(3)


In contrast, the Hsd17b2 mRNA quantity in GCs is not assumed to be strongly controlled by FSH [25] nor affected by EDCs, and the corresponding equation is simply:
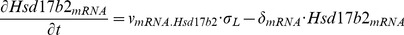
(4)


For the three enzymes *ε* (in pg/cell), the following mass-balance equation, with synthesis rate constant *ν_prot,ε_* (/min) and degradation rate constant *δ_prot_* (/min), applies:
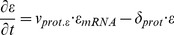
(5)Our experiments on GCs [20] gave us Hsd17b1 and Hsd17b2 initial mRNA and protein quantities, relative to Cyp19. We translated them to absolute values (pg/cell) on the basis of the initial quantities of Cyp19 mRNA and protein in GCs obtained from the literature (Table 1). We assumed that these values were steady-state values, in the absence of FSH stimulation, EDC alteration, or experimental variability. Values for the mRNA and protein degradation rate constants (*δ_mRNA,ε_* and *δ_prot,ε_*) were found in the literature (Table 2). Using the above steady-state assumption, we set equations 1, 2, and 4 for mRNA quantities, and equation 5 for protein quantities, equal to zero and rearranged them for *ν_mRNA.ε_* and *ν_prot.ε_*. The value of *ν_mRNA.ε_* was computed for the three enzymes *ε* as:

**Table 1:** Granulosa cell specific mRNA and protein initial values used.

Initial values	Name	Experimental data (ratio to aromatase)	Value (pg/cell)
Aromatase mRNA quantity	*Cyp19_mRNA_*	1	4.96×10^−8^ a
Hsd17b1 mRNA quantity	*Hsd17b1_mRNA_*	2.07	1.03×10^−7^ b
Hsd17b2 mRNA quantity	*Hsd17b2_mRNA_*	0.14	7.00×10^−9^ b
Aromatase protein quantity	*Cyp19*	1	0.1^c^
Hsd17b1 protein quantity	*Hsd17b1*	2.1	0.21^b^
Hsd17b2 protein quantity	*Hsd17b2*	0.14	0.014^b^

a Harada *et al.*, 1999 [45].

b Values obtained from our relative *in vitro* data and the absolute values found in the literature for aromatase (see text).

c Auvray *et al.*, 2002 [46].

**Table 2:** Model parameter values (for one cell) obtained from direct measurements on granulosa cells *in vitro* or from the published literature values.

Parameter (units)	Symbol	Value
mRNA degradation (/min)	*δ_mRNA_*	6.00×10^−3*a*^
protein degradation (/min)	*δ_prot_*	3.00×10^−3*a*^
Aromatase mRNA synthesis (pg/min)	*υ_mRNA.Cyp19_*	3.00×10^−10*b*^
Hsd17b1 mRNA synthesis (pg/min)	*υ_mRNA.Hsd_*_17*b*1_	6.00×10^−10*b*^
Hsd17b2 mRNA synthesis (pg/min)	*υ_mRNA.Hsd_*_17*b*2_	4.20×10^−11*b*^
Aromatase protein synthesis (/min)	*υ_prot.Cyp_*_19_	6000^b^
Hsd17b1 protein synthesis (/min)	*υ_prot.Hsd_*_17*b*1_	6300^b^
Hsd17b2 protein synthesis (/min)	*υ_prot.Hsd_*_17*b*2_	6000^b^
Maximal reaction rates *V_max_* (pmoles/min/pg enzyme)		
Hsd17b2, T → A reaction	*λ_Hsd_*_17*b*2,*T*_	6.65×10^−8*c*^
Hsd17b2, E_2_ → E_1_ reaction	*λ_Hsd_*_17*b*2,*E*2_	7.91×10^−8*c*^
Michaelis-Menten constants (pmoles)		
Hsd17b2, for T	*ξ_Hsd_*_17*b*2,*T*_	5.67×10^−6*c*^
Hsd17b2, for E_2_	*ξ_Hsd_*_17*b*2,*E*2_	5.40×10^−6*c*^
A extra- over intra-cellular partition coefficient (unitless)	*R_oi,A_*	0,0124^d^
T extra- over intra-cellular partition coefficient (unitless)	*R_oi,T_*	0,013^d^
E_1_ extra- over intra-cellular partition coefficient (unitless)	*R_oi,E_*_1_	0,0084^d^
E_2_ extra- over intra-cellular partition coefficient (unitless)	*R_oi,E_*_2_	0,0108^d^
A excretion rate constant (ml/min)	*K_out,A_*	1×10^−8*e*^
T excretion rate constant (ml/min)	*K_out,T_*	1×10^−8*e*^
E_1_ excretion rate constant (ml/min)	*K_out,E_*_1_	1×10^−8*e*^
E_2_ excretion rate constant (ml/min)	*K_out,E_*_2_	1×10^−8*e*^
Ovary blood flow (ml/min)	*F_ov_*	0.2654^f^
Individual granulosa cell volume (ml)	*V_GC_*	0.27×10^−9^ ml^g^

A, androstenedione; T, testosterone; E_1_, estrone; E_2_, estradiol.

a Hargrove, 1993a [47]; Hargrove, 1993b [48].

b mRNA and protein synthesis rates were calculated under steady-state assumption with data from direct measurements on granulosa cells *in vitro* (see text).

c Renwick *et al.*, 1981 [49].

d Breen *et al.*, 2009 [26].

e Data were arbitrately fixed.

f Plowchalk and Teeguarden, 2002 [50].

g Direct *in vitro* measurement.



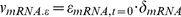
(6)Similarly, assuming that one mRNA gets translated into one protein, the value of *ν_prot.ε_* was computed for the three enzymes *ε* as:
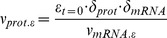
(7)


##### Steroid biotransformation

The relevant enzymatic reactions in GCs, catalyzed by Cyp19, Hsd17b1, and Hsd17b2, were modeled by the following competitive Michaelis-Menten metabolic terms *α_i_*, where *λ_ε,Z_* (pmoles/min/pg enzyme) and ξ*_ε,Z_* (pmoles) denote respectively *V_max_* and *K_m_* parameters for enzyme *ε* and substrate *Z* (A, T, E_1_, or E_2_).

Methoxychlor metabolite HPTE inhibits aromatase activity directly and competitively [20]. To model that effect, the parameter *f_M_* in equations 9 and 12 below represents the fold-change of the aromatase *K_m_* for its substrate *Z* (ξ*_Cyp19,Z_*), observed *in vitro*. Since aromatase activity is inversely proportional to its *K_m_*, this fold-change *f_M_* corresponds to the inverse of fold-change for aromatase enzymatic activity between treated and control cells. Fold-change for *K_m_* parameters ξ*_Cyp19,A_* and ξ*_Cyp19,T_* corresponds to:
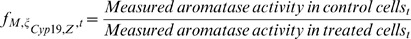
(8)


The conversion of A into E_1_ by aromatase takes into account T competition for the enzyme (the steroids are subscripted with *GC,* denoting the intra-cellular quantities):
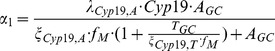
(9)


Conversion of A into T by Hsd17b1, with E_1_ competition:
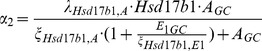
(10)


Conversion of T into A by Hsd17b2, with E_2_ competition:
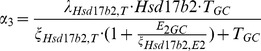
(11)


Conversion of T into E_2_ by aromatase, with A competition:
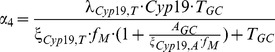
(12)


Conversion of E_1_ into E_2_ by Hsd17b1, with A competition:
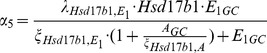
(13)


Conversion of E_2_ into E_1_ by Hsd17b2, with T competition:
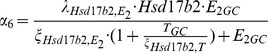
(14)


In order to model the isotopic measurement of tritiated water (T_2_O) production during the conversion of tritiated A to E_1_ (see *in vitro* experimental section), we need the formation rate of T_2_O, which is simply:
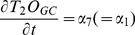
(15)


The parameters of the above equations are listed in Table 2.

#### Transport kinetics

The model was first developed to simulate *in vitro* conditions, and then adapted to model *in vivo* conditions. While the GC internal workings remained the same, different exchanges with the environment had to be described (Figure 2).

*Transport kinetics* in vitro. The *in vitro* model is divided in two compartments: GCs and culture medium (Figure 2A). For A (pmoles), T (pmoles), E_1_ (pmoles), E_2_ (pmoles), and FSH (pg), simple diffusion kinetics were assumed. The hormone quantity in a GC (*X_GC_*) has a rate of change equal to:
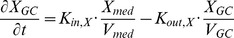
(16)where *K_in,X_* (ml/min) is the rate of medium (“med”) uptake by the GC, *K_out,X_* (ml/min) the rate of excretion by the GC, *X_med_* (pmoles or pg) the hormone quantity for one GC in the medium (total quantity divided by the number of cells used in a given assay), *V_med_* (ml) the volume of culture medium for one GC (total volume divided by the number of cells), and *V_GC_* (ml) the volume of one GC. *K_in,X_* was computed by dividing *K_out,X_* by the extra- over intra-cellular partition coefficient *R_oi,X_* (unitless), given in the literature [26]:



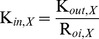
(17)Conversely, the hormone quantity for one cell in the medium (*X_med_*) has a rate of change equal to:
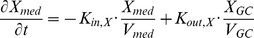
(18)


The cellular kinetics of A, T, E_1_, and E_2_ quantities depend on the entry in and exit from the cell and on their metabolism by Cyp19, Hsd17b1, or Hsd17b2:
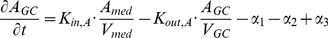
(19)

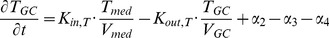
(20)

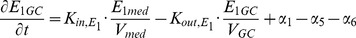
(21)

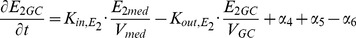
(22)where the *α_i_* are the Michaelis-Menten metabolic terms described in equations 9 to 14. The diffusion and transport of T_2_O in the *in vitro* system was not modeled, as the total quantity of T_2_O formed was directly measured.

*Transport kinetics* in vivo. For *in vivo* simulations (Figure 2B), the ovary was subdivided into three compartments: GCs, thecal and interstitial cells (“others”), and extracellular/vascular space (“ext”). The transport kinetics of hormone *X* in each cellular compartment depend on entry rate constant (*K_in_*) and exit rate constant (*K_out_*) for a cell, on the hormone concentrations in each cell, and on the number of cells (*N_GCs_* or *N_others_*). The differential equations for the “GC” and the “other cell” compartments are:
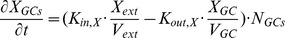
(23)

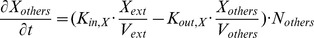
(24)where *V_ext_* and *V_others_* are the volumes of the extracellular and “other cell” compartments, respectively.

**Figure 3:**
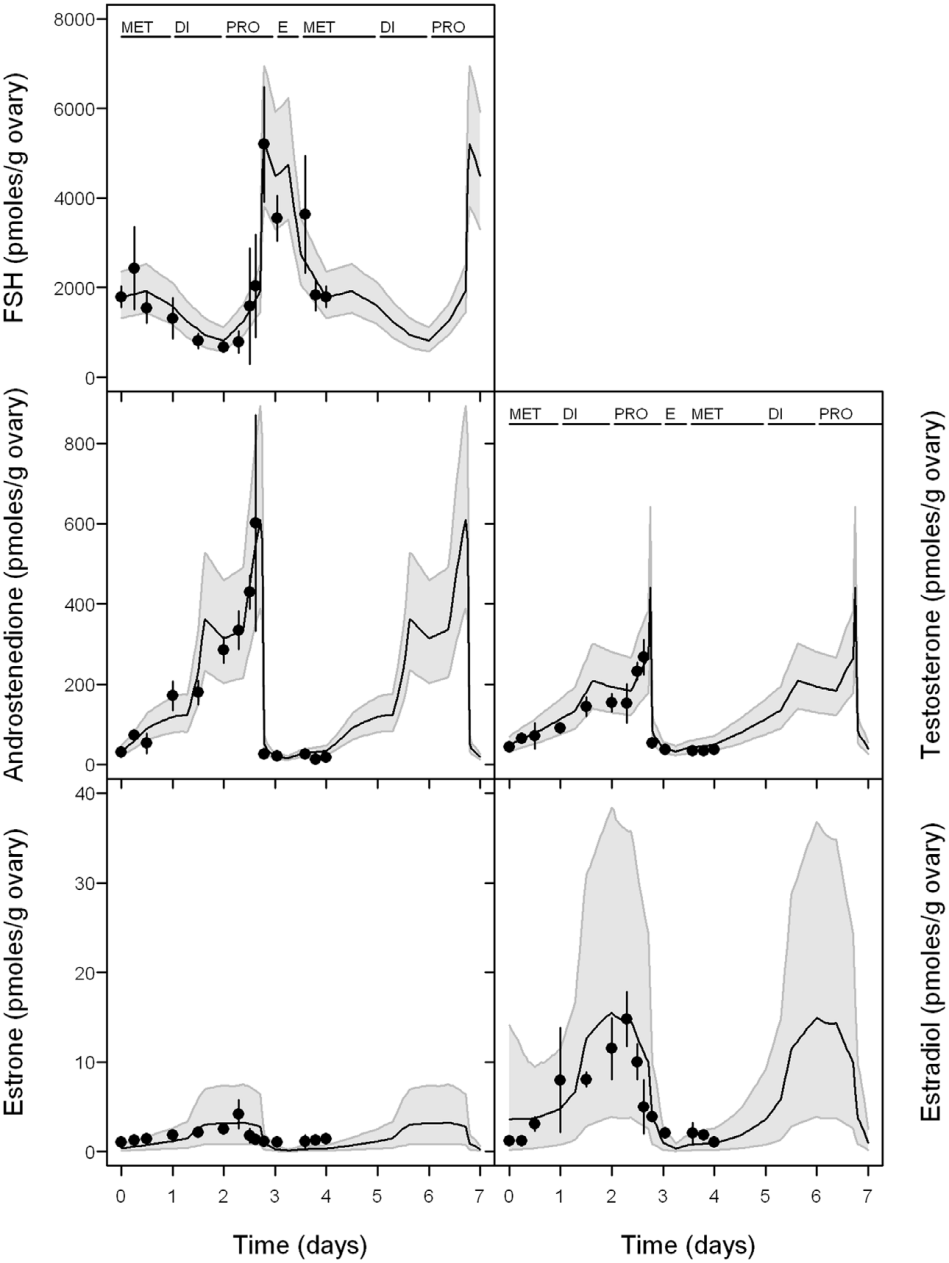
Experimental data *vs* predictions for FSH and sex steroid hormones in normal cycling rat. The black line represents mean model predictions with 95% confidence interval (grey band); points represent our experimental observations (mean of 10 measurements ± standard deviation).



The differential equation for quantity *X_ext_* in the extracellular compartment is:
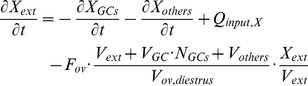
(25)where *Q_input,X_* (pmoles or pg/min) is the rate of input of hormone *X* in the ovary (coming from blood), *F_ov_* (ml/min) the efflux of *X* from the ovary (clearance by blood flow), and *V_ov,diestrus_* the ovarian volume at diestrus (which was set at 0.05 ml [27]). For mimicking the female estrus cycle *in vivo*, *Q_input,X_* for FSH and androgens were modeled as cyclic forcing functions, which were adjusted to give ovarian concentrations matching our *in vivo* physiological observations (see Figure 3). *Q_input,X_* is determined as:

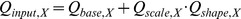
(26)where *Q_base,X_* (pmoles or pg/min) is the constant baseline concentration of hormone *X*, *Q_scale,X_* (unitless) the constant scale for hormone *X* magnitude, and *Q_shape,X_* (pmoles or pg/min) the variable magnitude of hormone *X* (adjusted to match the known hormone concentrations).

The time courses of *N_GCs_*, *V_ext_*, and *V_others_* during the estrous cycle were also modeled by forcing functions. The intracellular kinetic equations of the various hormones were the same as in the *in vitro* model (see metabolic reaction section).

#### Parameter value assignment and model calibration

Whenever possible, the model parameters were set to meaningful and physiologically based values that we directly measured in vitro or that we found in the published literature (Table 2).

The remaining model parameters (Table 3) were calibrated using *in vitro* experimental data that we developed ourselves (see above, *in vitro* data section), or that were published in the literature (Information S1). A Bayesian numerical approach, Markov Chain Monte Carlo (MCMC) simulations [28], was used.

**Table 3:** Prior distributions of the model parameters (for one granulosa cell) to be calibrated by MCMC sampling.

Parameter (units)	Symbol	Prior distribution
FSH effect on aromatase mRNA transcription (/pg FSH)	*κ_Cyp_*_19_	U (0, 1×10^7^)^a^
FSH effect on Hsd17b1 mRNA transcription (/pg FSH)	*κ_Hsd_*_17*b*1_	U (0, 1×10^6^)^a^
Maximal reaction rates *V_max_* (pmoles/min/pg enzyme)		
Aromatase		
A → E_1_ reaction	*λ_Cyp_*_19,*A*_	LN (1.33×10^−7^, 1.2)a,b,c,d
T → E_2_ reaction	*λ_Cyp_*_19,*T*_	LN (1.33×10^−7^, 2.0)^d^
Hsd17b1		
A → T reaction	*λ_Hsd_*_17*b*1,*A*_	LN (7.59×10^−8^, 2.0)e,f,g
E_1_ → E_2_ reaction	*λ_Hsd_*_17*b*1,*E*1_	LN (1.03×10^−5^, 2.0)e,f,g
Michaelis-Menten constants (pmoles)		
Aromatase		
For A	ξ*_Cyp_*_19,*A*_	LN (8.10×10^−9^, 1.2) a,b,c,d
For T	ξ*_Cyp_*_19,*T*_	LN (3.24×10^−8^, 2.0)^d^
Hsd17b1		
For A	ξ*_Hsd_*_17*b*1,*A*_	LN (4.32×10^−5^, 2.0) e,f,g
For E_1_	ξ*_Hsd_*_17*b*1,*E*1_	LN (5.29×10^−6^, 2.0) e,f,g
Mean inter-study random effect (arbitrary unit)	*µ*_0_	LN (1, 2.0)
Measurement variance for inter-study random effects	*Σ*_1_	HN (0.5)
Measurement variance for data likelihood of mRNA and proteins	*Σ*_2_	HN (0.2)
Measurement variance for data likelihood of hormone measurements	*Σ*_3_	HN (0.2)

LN (geometric mean, geometric SD): lognormal distribution; U (min, max): uniform distribution; HN (SD): halfnormal distribution with mean at zero.

Prior distribution for *V_max_* and *K_m_* parameters and for FSH effects are obtained and estimated from direct measurements on granulosa cells *in vitro*.

a Quignot *et al.*, 2012a [20].

b Odum *et al.*, 2001 [51].

c Auvray *et al.*, 2002 [46].

d Krekels *et al.*, 1990 [52].

e Ishikura *et al.*, 2006 [53].

f Renwick *et al.*, 1981 [49].

g Steckelbroeck *et al.*, 2003 [54].

The published *in vitro* data we used to calibrate the model included different cell pre-treatment protocols, which induced a large inter-study variability in baseline transcription rates *ν_mRNA.ε_*. That random effect was modeled with a variability factor *σ_L_* (see equations 1, 2, and 4), assumed to be log-normally distributed around a mean *μ_σ_*, with variance *Σ*_1_. The hyperparameters *μ_σ_* and *Σ*_1_ were in turn assigned vague prior distributions (Table 3). The individual random effects *σ_L_* (one per data set used, see Information S1), *μ_σ_*, and *Σ*_1_ were calibrated together with the other parameters.

The other parameters to be calibrated were assigned a prior distribution (Table 3). We mostly used lognormal distributions with geometric means set at physiologically relevant values. The geometric standard deviations were set to 2 or 1.2 for the parameters for which we had better information (Table 3). The data likelihoods were assumed to follow a lognormal distribution around the model predictions, a standard assumption with such measurements. The measurement error variances, which were assumed to be different between mRNA/protein quantities (*Σ*_2_) and hormone measurements (*Σ*_3_) (Table 3), were calibrated together with the other (physiological) parameters. A total of 24 parameters (11 physiological and 13 statistical) were MCMC sampled.

MCMC simulations (Metropolis-Hastings algorithm) were performed in triplicate chains of 20,000 iterations. For each model parameter sampled, convergence was evaluated using the last 10,000 iterations from each chain and the potential scale reduction criterion 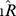 of Gelman and Rubin [29].

### Flux Analyses of *In Vitro* and *In Vivo* Experiments

Maximum *a posteriori* probability estimates of the calibrated parameters (Table 4) were used to do metabolic flux analyses [30], computing the rate of each steroid biotransformation reaction (*α*_1_ to *α*_6_, equations 9 to 14) as a function of time, to determine the predominant reactions for the conversion of A to E_2_.

**Table 4:** Summary statistics of the parameter posterior distributions after Bayesian calibration of the *in vitro* model.

Parameter	Average	SD	Maximum *a posteriori* probability estimates	0.5 percentile	2.5 percentile	97.5 percentile	99.5 percentile
*κ_Cyp_*_19_	2.08×10^6^	4.04×10^5^	2.08×10^6^	1.20×10^6^	1.36×10^6^	2.97×10^6^	3.27×10^6^
*κ_Hsd_*_17*b*1_	4.55×10^5^	1.76×10^5^	6.04×10^5^	1.08×10^5^	1.59×10^5^	8.51×10^5^	9.38×10^5^
*λ_Cyp_*_19,*A*_	1.04×10^−7^	1.78×10^−8^	1.07×10^−7^	6.54×10^−8^	7.36×10^−8^	1.44×10^−7^	1.59×10^−7^
*λ_Cyp_*_19,*T*_	3.72×10^−7^	2.16×10^−7^	2.67×10^−7^	8.39×10^−8^	1.15×10^−7^	9.61×10^−7^	1.33×10^−6^
*λ_Hsd_*_17*b*1,*A*_	1.03×10^−7^	7.86×10^−8^	5.65×10^−8^	1.30×10^−8^	2.12×10^−8^	3.14×10^−7^	4.76×10^−7^
*λ_Hsd_*_17*b*1,*E*1_	3.22×10^−5^	1.98×10^−5^	1.78×10^−5^	6.59×10^−6^	9.42×10^−6^	8.57×10^−5^	1.06×10^−4^
ξ*_Cyp_*_19,*A*_	8.32×10^−9^	1.53×10^−9^	8.25×10^−9^	5.16×10^−9^	5.7×10^−9^	1.17×10^−8^	1.32×10^−8^
ξ*_Cyp_*_19,*T*_	4.12×10^−8^	3.18×10^−8^	1.24×10^−8^	5.55×10^−9^	8.29×10^−9^	1.24×10^−7^	1.7×10^−7^
ξ*_Hsd_*_17*b*1,*A*_	6.49×10^−5^	4.93×10^−5^	4.84×10^−5^	9.58×10^−6^	1.38×10^−5^	1.98×10^−4^	2.74×10^−4^
ξ*_Hsd_*_17*b*1,*E*1_	2.91×10^−6^	1.96×10^−6^	1.30×10^−6^	4.74×10^−7^	6.61×10^−7^	8.00×10^−6^	1.12×10^−5^
*µ*_0_	0.407	0.163	1.46	0.131	0.177	0.81	1.08
*Σ*_1_	1.43	0.295	0.265	0.803	0.911	2.07	2.24
*σ_L_*_1_	1.07	2.1	0.117	0.0144	0.0367	5.42	19
*σ_L_*_2_	1.86	0.817	1.99	0.552	0.728	3.85	5.1
*σ_L_*_3_	0.0376	0.0181	0.0293	0.0109	0.0143	0.0839	0.116
*σ_L_*_4_	0.0314	0.0148	0.0193	0.00923	0.0122	0.0667	0.0938
*σ_L_*_5_	4.78	2.94	2.86	1.12	1.47	12.3	18.6
*σ_L_*_6_	0.028	0.0127	0.0213	0.00912	0.0111	0.0612	0.0808
*σ_L_*_7_	0.158	0.0306	0.153	0.0938	0.107	0.226	0.259
*σ_L_*_8_	0.696	0.422	0.612	0.129	0.188	1.75	2.48
*σ_L_*_9_	0.971	1.63	0.114	0.0182	0.0329	4.88	11.7
*σ_L_*_10_	0.685	0.101	0.685	0.46	0.506	0.916	1.03
*Σ*_2_	0.648	0.0842	0.626	0.47	0.503	0.832	0.901
*Σ*_3_	0.48	0.111	0.501	0.251	0.29	0.752	0.802

Each *σ_Li_* corresponds to the specific inter-study random effect *σ_L_* for each simulation set described in Information S1.

### Model Cross-validation Using *In Vivo* Data

In order to evaluate the predictive capacities of the model, we used random parameter vectors from their joint posterior distributions obtained by calibration with *in vitro* data (Table 4), and some other parameter distributions (Table 5), to simulate *in vivo* conditions. Table 5 includes parameters which were not calibrated from *in vitro* data because reasonable values were obtained for them in the literature, but which nonetheless have *in vivo* variability. We then simply compared the model predictions to the corresponding *in vivo* data. The cyclic entries of androgens and FSH in GCs and the time-varying number of ovarian cells were modeled as described in section “Transport kinetics *in vivo*”.

**Table 5:** Model parameter distributions used to describe *in vivo* variability (in addition to those of Table 4).

Parameter (units)	Symbol	Prior distribution
FSH dose rate: base concentration	*Q_base,FSH_*	LN (330, 1.2)
FSH dose rate: scale concentration	*Q_scale,FSH_*	LN (1450, 1.2)
A dose rate: base concentration	*Q_base,A_*	LN (1.2, 1.2)
A dose rate: scale concentration	*Q_scale,A_*	LN (18, 1.2)
T dose rate: base concentration	*Q_base,T_*	LN (3, 1.2)
T dose rate: scale concentration	*Q_scale,T_*	LN (13, 1.2)
Ovary blood flow (ml/min)	*F_ov_*	LN (0.2654, 1.1)
A excretion rate constant (ml/min)	*K_out,A_*	LN (1×10^−8^, 2.0)
A extra- over intra-cellular partition coefficient (unitless)	*R_oi,A_*	LN (0.0124, 1.2)
T excretion rate constant (ml/min)	*K_out,T_*	LN (1×10^−8^, 2.0)
T extra- over intra-cellular partition coefficient (unitless)	*R_oi,T_*	LN (0.0130, 1.2)
E_1_ excretion rate constant (ml/min)	*K_out,E_*_1_	LN (1×10^−8^, 2.0)
E_1_ extra- over intra-cellular partition coefficient (unitless)	*R_oi,E_*_1_	LN (0.0084, 1.2)
E_2_ excretion rate constant (ml/min)	*K_out,E_*_2_	LN (1×10^−8^, 2.0)
E_2_ extra- over intra-cellular partition coefficient (unitless)	*R_oi,E_*_2_	LN (0.0108, 1.2)
mRNA degradation (/min)	*δ_mRNA_*	LN (0.006, 1.2)
protein degradation (/min)	*δ_prot_*	LN (0.003, 1.2)
Aromatase mRNA synthesis (pg/min)	*υ_mRNA.Cyp19_*	LN (3×10^−10^, 1. 2)
Hsd17b1 mRNA synthesis (pg/min)	*υ_mRNA.Hsd_*_17*b*1_	LN (6×10^−10^, 1. 2)
Hsd17b2 mRNA synthesis (pg/min)	*υ_mRNA.Hsd_*_17*b*2_	LN (4.2×10^−11^, 1. 2)
Aromatase protein synthesis (/min)	*υ_prot.Cyp_*_19_	LN (6000, 1.2)
Hsd17b1 protein synthesis (/min)	*υ_prot.Hsd_*_17*b*1_	LN (6300, 1.2)
Hsd17b2 protein synthesis (/min)	*υ_prot.Hsd_*_17*b*2_	LN (6000, 1.2)
Maximal reaction rates *V_max_* (pmoles/min/pg enzyme)		
Hsd17b2, T → A reaction	*λ_Hsd_*_17*b*2,*T*_	LN (6.65×10^−8^, 2.0)
Hsd17b2, E_2_ → E_1_ reaction	*λ_Hsd_*_17*b*2,*E*2_	LN (7.91×10^−6^, 2.0)
Michaelis-Menten constants (pmoles)		
Hsd17b2, for T	*ξ_Hsd_*_17*b*2,*T*_	LN (5.67×10^−8^, 2.0)
Hsd17b2, for E_2_	*ξ_Hsd_*_17*b*2,*E*2_	LN (5.40×10^−6^, 2.0)

LN (geometric mean, geometric SD): lognormal distribution.

### Predictive Simulations of Endocrine Disruption

To evaluate the capacity of the above model to predict *in vivo* effects of EDCs on E_2_ secretion on the basis of *in vitro* data, we ran a series of simulations of endocrine disruption by atrazine, bisphenol A, methoxychlor metabolite HPTE, vinclozolin metabolite M2, and letrozole over two estrous cycles. The mRNA and *K_m_* fold-changes *f_X_* (equations 1–3, 8, 9, and 12) were changed to their experimentally observed values (see Table 6), starting eight hours after the beginning of the second modeled diestrus. We then compared the *in vivo* E_2_ quantities measured experimentally in EDC-treated females in diestrus with the model predictions. The hypothesis that the distributions of experimental data and model predictions were identical was statistically tested with a two-sample Kolmogorov-Smirnov test [31]. Differences with a *P* value of less than 0.05 were considered to be statistically significant.

**Table 6:** Modulation (fold-change) of steroidogenic enzymes mRNA levels and aromatase enzymatic activity following exposure of granulosa cells to selected chemicals.

Measurements	Atrazine	Bisphenol A	Methoxychlor metabolite HPTE	Vinclozolin metabolite M2	Letrozole
Direct aromatase enzymatic activity	0.99±0.11	0.94±0.14	0.89* ±0.11	0.98±0.09	0.29±0.10^a^
Aromatase mRNA levels	1.94* ±1.23	1.61* ±1.15	1.06±1.15	3.13* ±1.04	Not measured
Hsd17b1 mRNA levels	3.04* ±3.71	1.41±1.62	1.32±0.42	1.61* ±0.80	Not measured

Fold-changes: mean ± standard deviation.

* Statistically different from control, *p*<0.05.

a Odum *et al.*, 2002 [10].

### Software Used

Cell Designer 4.2 [32] was used to produce Figure 1. Model simulations, MCMC simulations for model calibration, and flux analyses were performed with GNU MCSim v5.4.0 [28]. Statistical analyses and plots were performed with R, version 2.14.0 [33].

## Results

### *In Vitro* Experimental Results

To evaluate and quantify how the selected EDCs affect aromatase and Hsd17b mRNA levels, as well as aromatase function, we exposed rat primary GCs (or microsomal fractions for direct aromatase activity) to atrazine, bisphenol A, methoxychlor metabolite HPTE, or vinclozolin metabolite M2. The chemical concentration used corresponded to the highest one found in rat ovaries following oral exposure to a high dose of each selected EDC [23]. None of the chemicals tested affected cell viability, as assessed with trypan blue exclusion staining and morphological evaluation. The purpose for measuring aromatase activity on microsomes (rather than in entire cells) was to discriminate a direct effect of chemicals at the functional protein level from an effect due to altered protein levels. Table 6 illustrates the fold-changes (relative to appropriate controls) for aromatase direct enzymatic activity, and aromatase and Hsd17b1 mRNA level modulation. In our experiments, Hsd17b2 mRNA levels were too low to be quantified. Atrazine, bisphenol A, and vinclozolin metabolite M2 did not affect aromatase direct activity, whereas HPTE decreased it by 11%.

Atrazine increased aromatase and Hsd17b1 mRNA levels with fold-inductions of 1.94 and 3.04, respectively. Bisphenol A increased 1.61-fold the amount of aromatase mRNA levels, but did not modify the Hsd17b1 mRNA levels. HPTE did not affect aromatase or Hsd17b1 mRNA levels. Vinclozolin up-regulated 3.13 and 1.61-fold aromatase and Hsd17b1 mRNA levels, respectively.

Experimental *in vitro* data for letrozole were found in the literature [10]. Hence, at the concentration of 50 nM, which corresponds to that found in rat ovaries after treatment with letrozole at 5 mg/kg, aromatase activity was decreased to 29% compared to control.

### *In Vivo* Experimental Results: Baseline Study

Gonadal sex steroid and blood FSH concentrations in healthy cycling female rats were measured at several times, falling in different periods of the estrous cycle (Figure 3). Those results are well in agreement with the published scientific literature [34].

### Model Calibration

Twenty-four model parameters were jointly calibrated using MCMC simulations. The three chain simulations converged after 10,000 iterations (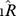 was at most 1.01 for all sampled parameters). The posterior fit after parameter Bayesian calibration has an average absolute deviation of 18.82% between data and predictions.

Table 4 presents summary statistics of the posterior distributions for the parameters calibrated. Those statistics are based on 30,000 iterations (the last 10,000 iterations from each of the three chains). For FSH effect on aromatase and Hsd17b1, while prior distributions were quite vague (see Table 3), posterior distributions indicated that the effect of FSH on aromatase is about four times higher than its effect on Hsd17b1. *V_max_* and *K_m_* for aromatase had informative priors and the posterior distributions were close to them; this probably may confirm the prior knowledge. However, although we used the same aromatase *V_max_* prior for A and T, posterior distributions revealed a 3-fold higher *V_max_* for T. On the contrary, according to posterior distributions, the aromatase *K_m_* for A is 5-fold smaller than the one for T. The posterior distributions for Hsd17b1 *V_max_* and *K_m_* were modified by a factor 1 to 2 for ξ*_Hsd_*_17*b*1,*E*1_, ξ*_Hsd_*_17*b*1,*A*_, and *λ_Hsd_*_17*b*1,*A*_, and by a factor 3 for *λ_Hsd_*_17*b*1,*E*1_. The average inter-study variability factor was about 40%. Study-specific variability factors ranged from 0.03 to about 5. The measurement error variances corresponded to a coefficient of variation of about 65% for mRNA/protein quantities (*Σ*_2_), and 48% for hormone measurements (*Σ*_3_).

### Flux Analyses of *In Vitro* and *In Vivo* Experiments

**Figure 4:**
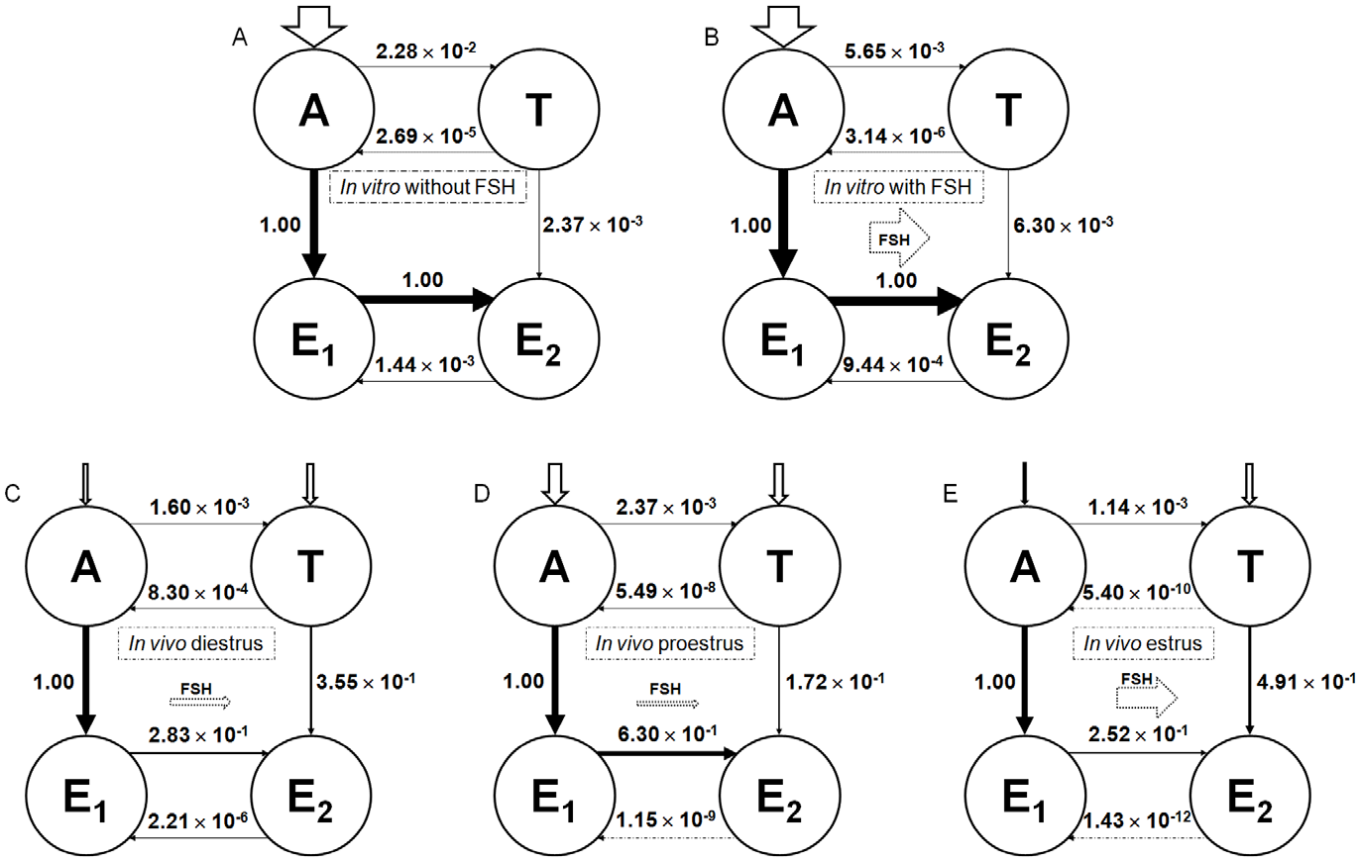
Flux analyses of *in vitro* and *in vivo* experiments. Graphs A and B represent the *in vitro* flux analysis of steroid hormones conversion at 48 h after addition of 200 nM A into the medium, without or with FSH 20 ng/ml. Graphs C, D, and E illustrate the *in vivo* flux analysis of steroid hormones conversion at several times of the estrus cycle (corresponding to diestrus, proestrus, and estrus stages). The aromatization reaction of A into E1 is taken as the reference reaction for each condition. The flux values for that reference were 7.29×10^−9^ pmoles/min/cell *in vitro* without FSH, 8.72×10^−8^ pmoles/min/cell *in vitro* with FSH, 6.09×10^−9^ pmoles/min/cell *in vivo* in the diestrus stage, 6.17×10^−9^ pmoles/min/cell in the proestrus stage, and 5.10×10^−9^ pmoles/min/cell in the estrus stage of the estrous cycle. Values for the other reactions in each condition are relative to the corresponding reference. Arrow thicknesses are proportional to the flux absolute values.

Figure 4A, B shows the results of A, E_1_, T, and E_2_ *in vitro* interconversion flux analysis 48 h after addition of the substrate A (200 nM), with or without FSH (20 ng/ml). The flux value for the reference reaction A to E_1_ increased from 7.29×10^−9^ pmoles/min/cell (without FSH) to 8.72×10^−8^ pmoles/min/cell (with FSH). The other reaction relative values show that the preferential pathway for E_2_ synthesis in GCs *in vitro* is conversion of A into E_1_, which is then converted into E_2_, both with or without FSH.

Figure 4C, D, E shows the results of steroid hormone *in vivo* interconversion flux analysis at different times of the estrous cycle. The flux value for the reference reaction A to E_1_ increased from 5.10×10^−9^ pmoles/min/cell at the estrus stage to 6.09×10^−9^ pmoles/min/cell at the diestrus stage and to 6.17×10^−9^ at the proestrus stage. Those *in vivo* results, which show a preferential pathway for E_2_ synthesis trough E_1_ conversion, itself coming from A, are in accordance with the *in vitro* ones. Some differences between *in vitro* and *in vivo* flux analyses can be noted, like the greater conversion of T to E_2_ *in vivo* than *in vitro*, or the relative importance of the retroconversions of T and E_2_ to A and E_1_, respectively, in *in vitro* experiments compared to *in vivo* ones.

### *In Vivo* Model Simulation

In order to evaluate the model accuracy, we set the GC model parameters *in vivo* to the values found by calibration with *in vitro* data. *In vivo* parameter uncertainty and variability were modeled by distributions of hormone inputs, clearances, mRNA/protein degradation and specific synthesis, and Hsd17b2 apparent kinetic constant parameters (Table 5). These distributions, used in inputs to Monte Carlo simulations, yielded predictive confidence intervals.

We compared the model-predicted ovarian steroid concentrations with the data from baseline experiments (Figure 3). The mean of our model predictions were within the 95% confidence interval of the model predictions. A quantitatively close profile for predicted data and experimental data was observed for E_2_, whereas the values for E_1_ in the diestrus stage were somewhat under experimental data. Profiles for FSH and androgens are shown for informative purpose, since they were constructed (using forcing functions) to match the observed profiles.

### *In Vivo* Experimental Results: EDC Study

**Figure 5:**
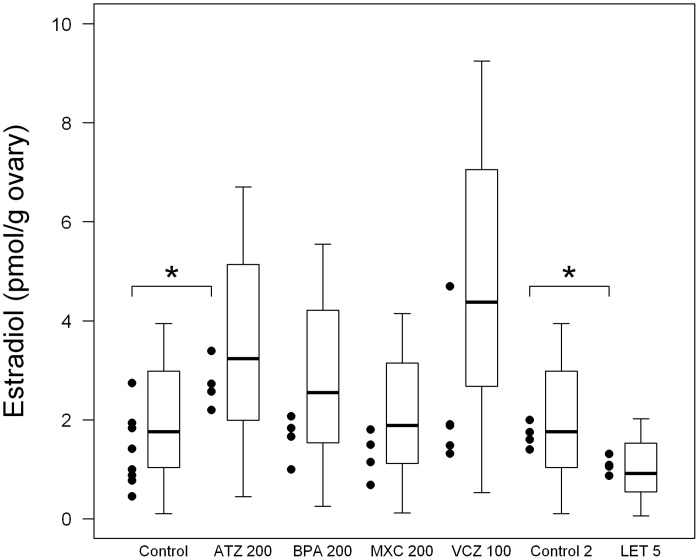
Experimental data *vs* predictions of estradiol levels in control and EDC-treated female rats at the diestrus stage. Experimental data are represented by points (n = 8 for control data, n = 4 for EDC-treated animals data). Statistical distributions of the model predictions are represented by boxplots (showing the distribution quartiles). Control is for atrazine 200 mg/kg, bisphenol A 200 mg/kg, and vinclozolin 100 mg/kg; control 2 is for letrozole 5 mg/kg. ATZ: atrazine; BPA: Bisphenol A; MXC: methoxychlor; VCZ: vinclozolin; LET: letrozole.

Figure 5 illustrates the distribution of experimentally measured ovarian E_2_ levels following EDC oral exposure. E_2_ levels were significantly increased in atrazine-treated females, whereas no statistically significant alteration of E_2_ was observed with bisphenol A, methoxychlor, and vinclozolin treatment. As far as vinclozolin-treated rats are concerned, one of those showed an elevated E_2_ ovarian concentration.

*In vivo* data extracted from the literature showed a significant decrease of E_2_ in letrozole-treated rat ovaries, compared to control [24].

### Predictions for Ovarian Estradiol Concentrations in EDC-treated Female Rats

*In vitro* results with atrazine, bisphenol A, methoxychlor metabolite HPTE, and vinclozolin metabolite M2 showed a modulation in mRNA levels after four hours of chemical exposure; and only cells treated with HPTE and letrozole showed a significant decrease in aromatase enzymatic activity (Table 6). To further evaluate the predictive capacity of the model, we simulated E_2_ concentrations in female rats exposed to atrazine, bisphenol A, methoxychlor, vinclozolin, and letrozole for six hours. After “*in vivo*” simulation with the mathematical model, we compared E_2_ values predicted with those experimentally measured (Figure 5). A two sample Kolmogorov-Smirnov test was performed for each pair of data (experimental versus predicted data for each treatment). It confirmed that the distributions of experimental data and model predictions were similar for control, atrazine, bisphenol A, methoxychlor, and letrozole treatments. Significantly different distributions were found only for vinclozolin treatment (*p = *0.021).

## Discussion

The model presented here offers a detailed description of some steroidogenic processes, focusing on what we felt to be the most important ones for *in vitro* to *in vivo* extrapolation. The Bayesian approach used for calibrating the model parameters permitted us to take into account both uncertainty and variability in experimental data, which is an asset for the relevance of the predictions. The *in vitro* and *in vivo* data we generated allowed us to finely calibrate and cross-validate the model, which was able to quantitatively predict E_2_ ovarian concentration in physiological conditions or after exposure to selected EDCs. This model, in spite of its limitations, has many potential mechanistic or predictive applications, as we discuss in the following.

### Model Development

In the context of EDC toxicity assessment, some authors developed systems biology models of the hypothalamic-pituitary-gonadal (HPG) axis. Many of them are graphical systems models, which allow researchers to visualize and think more clearly about the impact of chemicals on the HPG axis (as reviewed by [35], [36], [37]. They can also provide a framework for integration of quantitative computational models, such as those of Breen et al. [38], Watanabe et al. [39], and Li et al. [40]. Breen et al. [38] proposed a steady-state model of fathead minnow ovarian synthesis and release of T and E_2_; Watanabe et al.’s model [39] simulates synthesis and feedback loops for T, 11-ketotestosterone, E_2_, and vitellogenin plasma concentrations in male fathead minnow; Li et al.’s model [40] simulate E_2_, T, and vitellogenin plasma concentrations in female fathead minnow. Those models focused on fish as a target species since endocrine disruption is well documented in aquatic species [41]. However, the assessment of EDC toxicity for humans warrants the development of mammalian models. We chose to develop a computational model focusing on the last steps of steroidogenesis in the rat ovary. This choice seemed to be a good compromise between our purpose (make quantitative in vivo predictions for a mammal based on in vitro measurements), and the data available to calibrate and cross-validate our model.

### Model Calibration

The calibration of the model was done on the basis of several *in vitro* data sets, including our own. The diversity of protocols, in particular for cell pre-treatment, led us to model inter-study variability. Experiments reported in the literature were done to compare treatments with control conditions rather than to develop a computational model. For that reason, they lack endpoints such as time-response curves at several FSH levels, precursor hormone measurements, etc. In that sense, to develop a quantitative computational model forces one to identify the kind of data needed. Beyond answering the questions raised when developing the model, such a refinement of experimental design may yield new findings about cellular biology and toxicology *in vitro*. In any case, the model was able to account for the differences between studies and predicted the endpoints reasonably well. That can be considered as the first part of our model validation process.

### Model Evaluation with Cross-validation

Results from a baseline in vivo study, without EDCs, were used to evaluate our model ability to predict some features of steroid synthesis in normal physiological conditions. We used model parameter values estimated by calibration of the in vitro data “as is”, without adjustment, to simulate E_1_ and E_2_ production by the ovary in vivo. The results showed that the model was able to accurately simulate ovarian E_2_ concentrations during normal cycling in female rats. The results for E_1_ were less convincing, in particular during diestrus. We did not go as far as to model the ovarian steroid output, plasma concentrations, and the hypothalamic-pituitary (HP) feedback. That was beyond the scope of our work, but more importantly, modeling steroid output from the ovaries and sex steroid plasma concentrations would have required calibration of several more parameters and compromised the mere feasibility of model cross-validation.

### Model Predictions and Biological Insight in Baseline Conditions

Updating the *a priori* parameter distributions into posteriors gives us some insight into features of the rat sex steroid synthesis network. For example, the preferred conversion of A into E_1_ by aromatase (in spite of its conversion into T by Hsd17b1) seems due to differences in *K_m_* values of androstedione for aromatase and Hsd17b1, rather than to differences in *V_max_* values.

The flux analyses indicate that the preferential pathway for E_2_ synthesis involves E_1_ both *in vitro* and *in vivo*. They also point out the need to perform toxicity testing experiments under FSH-controlled conditions.

Flux analyses show clear differences between *in vitro* and *in vivo* conditions. For example, steroid inactivation reaction fluxes (T to A and E_2_ to E_1_) are ranged from 10^−3^ to 10^−6^ pmoles/min/cell *in vitro*, and ranged from 10^−4^ to 10^−12^ pmoles/min/cell in *in vivo* conditions. Those differences can be explained by differences in hormone inputs to the system. Fluxes depend on reaction parameter values and hormone inputs applied. We showed that keeping parameter values equal *in vitro* and *in vivo*, and simply changing hormone inputs, is enough to explain flux differences between *in vitro* and *in vivo* conditions.

### Model Predictive Capacity Evaluation with Selected EDCs

To further evaluate the model predictive capacity, we simulated *in vivo* steroid concentrations in the ovaries after chemical exposure and compared them to original experimental results. Simulations were performed by modifying aromatase *K_m_* or mRNA levels on the basis of transcriptomic and enzymatic activity data obtained *in vitro* for GCs. We limited our predictions to six hours post-exposure, a period during which feedback regulation can be assumed to be negligible.

Results show that our model predictive capacity was different according to treatment. Model predictions were found to follow the same distributions as the experimental data, except for vinclozolin. However, Figure 5 shows nuances between treatments. The model predicted reasonably well the early ovarian response in E_2_ concentration for adult female rats exposed to atrazine and letrozole. Atrazine and letrozole mechanisms of action can explain why their effects were the most clearly seen experimentally and the best predicted by the model after a few hours. Indeed, we have previously shown [27] that elevated aromatase mRNA expression (see also Table 6) and the subsequent increase in aromatase catalytic activity in atrazine-treated females explain a large part of the increase in estrogen levels. As far as letrozole is concerned, it was designed to be a specific aromatase inhibitor. The early ovarian responses in E_2_ concentration for adult female rats exposed to bisphenol A, methoxychlor, or vinclozolin were less well predicted. The effects of bisphenol A, HPTE, or vinclozolin M2 on aromatase or Hsd17b1 did not explain the *in vivo* modulation of estrogen levels following treatment, although they can significantly affect enzyme mRNA levels *in vitro.* Instead we previously hypothesized that the main mechanisms of action are: a disruption of the hypothalamic-pituitary-adrenal axis for methoxychlor and vinclozolin; a peripheral effect on conjugation/deconjugation metabolism processes for bisphenol A [27]. The model, which doesn’t predict very well variations of E_2_ concentrations following exposure to those three chemicals, may confirm that the effects on granulosa steroidogenesis are not predominant. Furthermore, vinclozolin predictions were less precise, and showed higher variability. That is actually an interesting feature: vinclozolin mechanism of action is known to be more complex, acting notably by its anti-androgenic metabolite M2 [4], and subject to variable amplification in the steroidogenesis pathway. The experimental data themselves showed higher variability for vinclozolin, although the small number of animals tested precludes strong conclusions.

Even if predictions for E_2_ levels compared well with experimental values, the usefulness of the model could be improved. First, it does not account for EDC effects on androgen precursors, and can only predict effects for chemicals that act on the last steps of steroidogenesis. An improvement would be to add other pathways to the mathematical model, such as steroidogenic processes in thecal cells. The model may also integrate effects on steroid receptors, like the estrogen one, which is the target of numerous chemicals [42]. The model also lacks numerous feedbacks, in particular those mediated by the HP axis. Thereby, for now, the model predictions for steroid ovarian concentrations are of limited value for a complete analysis of endocrine disruption. Rat HP axis feedback models previously described [43], [44] might be useful for coupling with ours.

### Model Potential

Despite the limitations discussed above, the model perspectives are multiple. All the reaction parameters can be modulated to reflect changes observed *in vitro*, for example. That approach can be very useful for investigating mixture and chronic effects. It can also help formulate hypotheses and design experiments aimed at understanding the mechanisms of endocrine toxicity, notably for the effects which follow a non-monotonic dose-response, like those of EDCs. A model integrating feedback regulations would permit to describe further targets, such as the HPG axis, enzyme inhibition, or local gene expression effects.

Observations of alterations in ovarian functions at molecular and biochemical levels are useful for regulatory decision-making only if these changes can be translated into effects at higher biological levels of organization. The model is able to make quantitative predictions about steroid secretion based on data on the impact of chemicals on the last steps of ovarian steroidogenesis. Sex steroid concentration changes, even of low scale, account for a large part of effects in reproductive toxicology, but it is not sufficient. Integrated models, predicting multiple endpoints relevant for reproductive toxicology assessment, have been developed in the fathead minnow [39], [40]. Since links between sex steroid concentration changes and reproductive toxicity are not clear in mammals, some work still has to be done.

### Conclusions

The model developed was able to predict a very sensitive and integrative reproductive endpoint: ovarian sex steroid levels, from *in vitro* data. The results of flux analyses and predictions of EDC-treated females show that the model not only fits the data empirically, but also captures major features of the GC steroidogenesis network. We carefully limited the scope of our model to ovarian secretion in order to be able to cross-validate it with the data available. In some cases, investigating effects simply on gonads can be a powerful tool for understanding whole-body hormone disruption, in which case the model might be a valuable tool for toxicity assessment. While the predictive capacity of this mathematical model is still limited, it already has potential applications for improved evaluation of endocrine disruption following chemical exposure, in particular for low levels and mixtures of pollutants.

## Supporting Information

Information S1 ***In vitro*** **data used for parameter estimation.** The table presents the endpoints experimentally measured by authors that allowed us to update our prior information. Data obtained are both from non-treated cells or FSH-induced conditions. All conditions and measurements were scaled down to one cell by dividing by the number of cells introduced in the assay system.
(DOC)
