# Supplementary material for: A Computational Model to Predict Rat Ovarian Steroid Secretion from In Vitro Experiments with Endocrine Disruptors
Source: PLoS One. 2013 Jan 11;8(1):e53891. doi: 10.1371/journal.pone.0053891 (PMC3543310; doi:10.1371/journal.pone.0053891)
Supplement: Information S1 — In vitro data used for parameter estimation. The table presents the endpoints experimentally measured by authors that allowed us to update our prior information. Data obtained are both from non-treated cells or FSH-induced conditions. All conditions and measurements were scaled down to one cell by dividing by the number of cells introduced in the assay system. (DOC) [file pone.0053891.s001.doc]

Supporting information S1. *In vitro* data used for parameter estimation.

The table presents the endpoints experimentally measured by authors which allowed us to update our prior information. Data obtained are both from non-treated cells or FSH-induced conditions. All conditions and measurements were scaled down to one cell by dividing by the number of cells introduced in the assay system.

| **Reference** | **Study number** | **Experimental conditions (assay volume and hormone quantities applied *per cell*)** | **Data (fold-changes or quantities *per cell*)** | **Simulation number** |
| --- | --- | --- | --- | --- |
| Inaoka, 2008 [1] | 1 | V_med_ = 1 x 10^-6^ ml  FSH = 0.03 pg | Fold induction (FSH treated over control): Cyp19 mRNA = 45 at 60 min, 10 at 720 min, 50 at 1440 min, 240 at 2880 min | 1 |
| Timossi, 2000 [2] | 2 | V_med_ = 4 x 10^-6^ ml  A = 0.004 pmol  FSH = 0 pg | E_2_ = 2.94 x 10^-5^ pmol at 2880 min | 2 |
|  |  | V_med_ = 4 x 10^-6^ ml  A = 0.004 pmol FSH = 2.4 x 10^-3^ pg | E_2_ = 5.87 x 10^-5^ pmol at 2880 min | 3 |
|  |  | V_med_ = 4 x 10^-6^ ml  A = 0.004 pmol FSH = 2.4 x 10^-2^ pg | E_2_ = 4.99 x 10^-4^ pmol at 2880 min | 4 |
| Nakamura, 2011 [3] | 3 | V_med_ = 2 x 10^-6^ ml  A = 2 x 10^-4^ pmol FSH = 0 pg | E_2_ = 5.40 x 10^-7^ pmol at 2880 min | 5 |
|  |  | V_med_ = 2 x 10^-6^ ml  A = 2 x 10^-4^ pmol FSH = 6 x 10^-2^ pg | E_2_ = 6.74 x 10^-6^ pmol at 2880 min | 6 |
| Lu, 2009 [4] | 4 | V_med_ = 1 x 10^-6^ ml  A = 1 x 10^-4^ pmol FSH = 0 pg | E_2_ = 1.30 x 10^-7^ pmol at 1440 min | 7 |
|  |  | V_med_ = 1 x 10^-6^ ml  A = 1 x 10^-4^ pmol FSH = 2 x 10^-2^ pg | E_2_ = 9.90 x 10^-7^ pmol at 1440 min | 8 |
| Chabrolle, 2008 [5] | 5 | V_med_ = 4 x 10^-6^ ml  A = 4 x 10^-4^ pmol FSH = 0 pg | E_2_ = 1.60 x 10^-4^ pmol at 2880 min | 9 |
|  |  | V_med_ = 4 x 10^-6^ ml  A = 4 x 10^-4^ pmol FSH = 0.01 pg | E_2_ = 3.70 x 10^-4^ pmol at 2880 min | 10 |
| Tamura, 2007 [6] | 6 | V_med_ = 2.5 x 10^-6^ ml  A = 2.62 x 10^-4^ pmol FSH = 0 pg | E_2_ = 1.50 x 10^-7^ pmol at 1440 min | 11 |
|  |  | V_med_ = 2.5 x 10^-6^ ml  A = 2.62 x 10^-4^ pmol FSH = 6.25 x 10^-2^ pg | E_2_ = 9.75 x 10^-7^ pmol at 1440 min | 12 |
| Tinfo, 2011 [7] | 7 | V_med_ = 8 x 10^-7^ ml  A = 1.6 x 10^-4^ pmol FSH = 0 pg | E_2_ = 1.92 x 10^-7^ pmol at 60 min | 13 |
|  |  | V_med_ = 8 x 10^-7^ ml  A = 1.6 x 10^-4^ pmol FSH = 0 pg | H_2_O = 0.88 x 10^-7^ pmol at 60 min | 14 |
| Kaminsky, 1997 [8] | 8 | V_med_ = 5 x 10^-6^ ml  E_1_ = 5 x 10^-4^ pmol FSH = 0 pg | E_2_ = 3.0 x 10^-5^ pmol at 60 min | 15 |
|  |  | V_med_ = 5 x 10^-6^ ml  E_1_ = 5 x 10^-4^ pmol FSH = 8.5 x 10^-4^ pg | E_2_ = 5.0 x 10^-5^ pmol at 60 min | 16 |
|  |  | V_med_ = 5 x 10^-6^ ml  E_1_ = 5 x 10^-4^ pmol FSH = 2 x 10^-3^ pg | E_2_ = 11.0 x 10^-5^ pmol at 60 min | 17 |
|  |  | V_med_ = 5 x 10^-6^ ml  E_1_ = 5 x 10^-4^ pmol FSH = 4.15 x 10^-3^ pg | E_2_ = 12.0 x 10^-5^ pmol at 60 min | 18 |
|  |  | V_med_ = 5 x 10^-6^ ml  E_1_ = 5 x 10^-4^ pmol FSH = 8.3 x 10^-3^ pg | E_2_ = 17.5 x 10^-5^ pmol at 60 min | 19 |
|  |  | V_med_ = 5 x 10^-6^ ml  E_1_ = 5 x 10^-4^ pmol FSH = 2.08 x 10^-2^ pg | E_2_ = 19.0 x 10^-5^ pmol at 60 min | 20 |
|  |  | V_med_ = 5 x 10^-6^ ml  E_1_ = 5 x 10^-4^ pmol FSH = 4.17 x 10^-2^ pg | E_2_ = 23.0 x 10^-5^ pmol at 60 min | 21 |
| Zachow, 2000 [9] | 9 | V_med_ = 5 x 10^-6^ ml  A = 5 x 10^-4^ pmol FSH = 0 pg | Fold induction (FSH treated over control): Cyp19 mRNA = 1 at 2880 min  Hsd17b1 mRNA = 1 at 2880 min | 22 |
|  |  | V_med_ = 5 x 10^-6^ ml  A = 5 x 10^-4^ pmol FSH = 15 x 10^-4^ pg | Fold induction (FSH treated over control): Hsd17b1 mRNA = 2 at 2880 min | 23 |
|  |  | V_med_ = 5 x 10^-6^ ml  A = 5 x 10^-4^ pmol FSH = 15 x 10^-3^ pg | Fold induction (FSH treated over control): Cyp19 mRNA = 5 at 2880 min  Hsd17b1 mRNA = 2.5 at 2880 min | 24 |
|  |  | V_med_ = 5 x 10^-6^ ml  A = 5 x 10^-4^ pmol FSH = 15 x 10^-2^ pg | Fold induction (FSH treated over control): Hsd17b1 mRNA = 2 at 2880 min | 25 |
| Quignot, 2012 [10] | 10 | V_med_ = 3.33 x 10^-6^ ml  FSH = 0.067 pg | Cyp19 mRNA = 4.96 x 10^-8^ pg at 0 min, 6.24 x 10^-8^ pg at 240 min Hsd17b1 mRNA = 1.03 x 10^-7^ pg at 0 min, 1.21 x 10^-7^ pg at 240 min | 26 |
|  |  | V_med_ = 3.33 x 10^-6^ ml  FSH = 0.067 pg | Cyp19 mRNA = 4.96 x 10^-8^ pg at 0 min, 6.02 x 10^-8^ pg at 240 min Hsd17b1 mRNA = 1.03 x 10^-7^ pg at 0 min, 1.20 x 10^-7^ pg at 240 min | 27 |
|  |  | V_med_ = 3.33 x 10^-6^ ml  FSH = 0.067 pg | Cyp19 mRNA = 4.96 x 10^-8^ pg at 0 min, 5.78 x 10^-8^ pg at 240 min Hsd17b1 mRNA = 1.03 x 10^-7^ pg at 0 min, 1.19 x 10^-7^ pg at 240 min | 28 |
|  |  | V_med_ = 3.33 x 10^-6^ ml  FSH = 0.067 pg | Cyp19 mRNA = 4.96 x 10^-8^ pg at 0 min, 5.65 x 10^-8^ pg at 240 min Hsd17b1 mRNA = 1.03 x 10^-7^ pg at 0 min, 1.11 x 10^-7^ pg at 240 min | 29 |
|  |  | V_med_ = 3.33 x 10^-6^ ml  FSH = 0.067 pg | Cyp19 mRNA = 4.96 x 10^-8^ pg at 0 min, 2.09 x 10^-7^ pg at 240 min Hsd17b1 mRNA = 1.03 x 10^-7^ pg at 0 min, 4.05 x 10^-7^ pg at 240 min | 30 |
|  |  | V_med_ = 3.33 x 10^-6^ ml  FSH = 0.067 pg | Cyp19 mRNA = 4.96 x 10^-8^ pg at 0 min, 2.01 x 10^-7^ pg at 240 min Hsd17b1 mRNA = 1.03 x 10^-7^ pg at 0 min, 4.03 x 10^-7^ pg at 240 min | 31 |
|  |  | V_med_ = 3.33 x 10^-6^ ml  FSH = 0.067 pg | Cyp19 mRNA = 4.96 x 10^-8^ pg at 0 min, 1.93 x 10^-7^ pg at 240 min Hsd17b1 mRNA = 1.03 x 10^-7^ pg at 0 min, 3.99 x 10^-7^ pg at 240 min | 32 |
|  |  | V_med_ = 3.33 x 10^-6^ ml  FSH = 0.067 pg | Cyp19 mRNA = 4.96 x 10^-8^ pg at 0 min, 1.89 x 10^-7^ pg at 240 min Hsd17b1 mRNA = 1.03 x 10^-7^ pg at 0 min, 3.72 x 10^-7^ pg at 240 min | 33 |

V_med_: volume of culture medium for one cell; FSH: FSH quantity for one cell in the culture medium; A: Androstenedione quantity for one cell in the culture medium; T: Testosterone quantity for one cell in the culture medium; E_1_: Estrone quantity for one cell in the culture medium; E_2_: Estradiol quantity for one cell in the culture medium

**References**

1. Inaoka Y, Yazawa T, Mizutani T, Kokame K, Kangawa K, et al. (2008) Regulation of P450 oxidoreductase by gonadotropins in rat ovary and its effect on estrogen production. Reprod Biol Endocrinol 6: 62.

2. Timossi CM, Barrios-de-Tomasi J, Gonzalez-Suarez R, Arranz MC, Padmanabhan V, et al. (2000) Differential effects of the charge variants of human follicle-stimulating hormone. J Endocrinol 165: 193-205.

3. Nakamura E, Otsuka F, Inagaki K, Miyoshi T, Matsumoto Y, et al. (2011) Mutual regulation of growth hormone and bone morphogenetic protein system in steroidogenesis by rat granulosa cells. Endocrinology 153: 469-480.

4. Lu C, Yang W, Chen M, Liu T, Yang J, et al. (2009) Inhibin A inhibits follicle-stimulating hormone (FSH) action by suppressing its receptor expression in cultured rat granulosa cells. Mol Cell Endocrinol 298: 48-56.

5. Chabrolle C, Jeanpierre E, Tosca L, Rame C, Dupont J (2008) Effects of high levels of glucose on the steroidogenesis and the expression of adiponectin receptors in rat ovarian cells. Reprod Biol Endocrinol 6: 11.

6. Tamura K, Matsushita M, Endo A, Kutsukake M, Kogo H (2007) Effect of insulin-like growth factor-binding protein 7 on steroidogenesis in granulosa cells derived from equine chorionic gonadotropin-primed immature rat ovaries. Biol Reprod 77: 485-491.

7. Tinfo NS, Hotchkiss MG, Buckalew AR, Zorrilla LM, Cooper RL, et al. (2011) Understanding the effects of atrazine on steroidogenesis in rat granulosa and H295R adrenal cortical carcinoma cells. Reprod Toxicol 31: 184-193.

8. Kaminski T, Akinola L, Poutanen M, Vihko R, Vihko P (1997) Growth factors and phorbol-12-myristate-13-acetate modulate the follicle-stimulating hormone- and cyclic adenosine-3',5'-monophosphate-dependent regulation of 17beta-hydroxysteroid dehydrogenase type 1 expression in rat granulosa cells. Mol Cell Endocrinol 136: 47-56.

9. Zachow RJ, Ramski BE, Lee H (2000) Modulation of estrogen production and 17beta-hydroxysteroid dehydrogenase-type 1, cytochrome P450 aromatase, c-met, and protein kinase Balpha messenger ribonucleic acid content in rat ovarian granulosa cells by hepatocyte growth factor and follicle-stimulating hormone. Biol Reprod 62: 1851-1857.

10. Quignot N, Desmots S, Barouki R, Lemazurier E (2012) A comparison of two human cell lines and two rat gonadal cell primary cultures as in vitro screening tools for aromatase modulation. Toxicol In Vitro 26: 107-118.
